# Design and Development of Rehabi, a mHealth Telerehabilitation Platform with Markerless Motion Analysis

**DOI:** 10.3390/bioengineering13030308

**Published:** 2026-03-06

**Authors:** Arturo González-Mendoza, Hipólito Aguilar-Sierra, Rafael Zepeda-Mora, Aldo Alessi-Montero, Gerardo Rodríguez-Reyes, Lidia Núñez Carrera, Ivett Quiñones-Uriostegui, Paola Ayala-Cadena, Adriana Gomez-Verdad

**Affiliations:** 1 National Institute of Rehabilitation (INR), Mexico City 14389, Mexico; rafael_zepeda_mora@hotmail.com (R.Z.-M.); aldoalessi@gmail.com (A.A.-M.); grodriguezreyes@gmail.com (G.R.-R.); lidianc2012@gmail.com (L.N.C.); iquinonesu@gmail.com (I.Q.-U.); paoareli2012@gmail.com (P.A.-C.); schneejpc@gmail.com (A.G.-V.); 2 Department of Engineering, Faculty of Engineering, Universidad La Salle Mexico, Condesa Campus, Mexico City 06140, Mexico; hipolito.aguilar@lasalle.mx

**Keywords:** telerehabilitation, mobile health, markerless motion analysis, user-centered design, clean software architecture

## Abstract

Musculoskeletal disorders such as rheumatoid arthritis and osteoarthritis affect millions worldwide and are projected to rise sharply by 2050, highlighting the importance of scalable telerehabilitation. This paper introduces Rehabi, a mobile, user-friendly tele-rehabilitation platform that centrally integrates markerless motion for biomechanical assessment and monitoring. Rehabi development followed a user-centered methodology, combining questionnaires, interviews, and natural language processing to elicit requirements from patients and clinicians. The system architecture was implemented in accordance with Clean Architecture principles to ensure modularity and scalability. In a pilot clinical validation of the markerless motion algorithm integrated into Rehabi, 14 post-arthroplasty patients showed moderate agreement for hip flexion (ICC = 0.686) and good agreement for knee flexion (ICC = 0.801). Although the sample was small, the results show a promising trend suggesting that mobile markerless motion capture may be a viable option for remote assessment and monitoring.

## 1. Introduction

Telemedicine is defined as the delivery of healthcare services where distance is a critical factor, using information and communication technologies (ICTs) for diagnosis, treatment, prevention, and patient education [1,2,3]. The COVID-19 pandemic prompted the adoption of telemedicine, demonstrating its value for continuity of care and expanding access [1,4,5,6,7,8]. Telemedicine encompasses several modalities [1,2,9]: these include teleconsulting (remote medical consultations via video or audio platforms) [10], telemonitoring (the continuous assessment of patients through audio, video, questionnaires, or other electronic information systems) [11], and telerehabilitation (the delivery of rehabilitation services at a distance) [2,6,8].

Within telemedicine, due to advantages such as remote evaluation, progress monitoring, and individualized supervised exercise at home [2,6,8], telerehabilitation is gaining prominence. This approach is particularly beneficial for musculoskeletal disorders like rheumatoid arthritis and osteoarthritis, which affected more than 15 million and 500 million people worldwide, respectively, by 2020, and are projected to rise sharply by 2050, driven by population growth and aging [12,13]. Evidence of clinical impact, however, remains mixed: while systematic reviews and randomized trials report benefits, other studies highlight heterogeneity in protocols and sensitivity to moderators such as patient selection, digital literacy, capture conditions, connectivity, device quality, and clinician training [14,15,16].

Traditionally, telerehabilitation has been delivered via web-based platforms that centralize data management and enable clinician-led sessions, but telerehabilitation has shifted toward mobile Health (mHealth) delivery [17,18,19,20]. In parallel with mHealth, physical education is embracing digital transformation. Guided by UNESCO’s call for balanced and sustainable technology integration, it emphasizes motor skill development, physical activity, and health promotion [16]. Leveraging Artificial Intelligence (AI) algorithms and ICT tools as wearable sensors, these approaches increasingly incorporate motion-tracking (the process of capturing, analyzing, and quantifying human movement) solutions that seek to enhance physical education and therapy through personalized exercise, real-time feedback, and progress tracking [16,17,21]. Within this context, markerless motion capture systems have emerged as a practical alternative to traditional marker-based systems, reducing setup time, cost, and patient discomfort while preserving natural movement. Comparative studies show strong agreement between markerless motion and marker-based motion for spatiotemporal metrics such as goniometry, walking speed, step time, and step length (ICC 0.81–0.98), supporting their reliability for routine assessment [22,23,24].

Recent developments illustrate recurring technical approaches and challenges in telerehabilitation. Modular architectures decouple sensing hardware from analytics and interfaces, enabling scalable, device-agnostic deployment [25]. Vision-based applications demonstrate feasibility for home-based mobility assessment without specialized hardware, though they remain sensitive to capture conditions and require standardized protocols [26]. IoT and edge-to-cloud prototypes integrate physiological sensors with local preprocessing to support resilience in low-connectivity environments [27]. Mobile apps increasingly combine passive sensing, standardized questionnaires, and conversational agents to improve adherence and data quality [28]. At the same time, policy-oriented reviews emphasize governance, workforce training, and cybersecurity as prerequisites for safe scale-up [29].

Taken together, these contributions clarify five recurring patterns: modularity for rapid adaptation, standardized capture for reliable vision-based measures, edge/cloud splits for resilience, conversational onboarding for adherence, and governance for safe scale-up. These insights inform the objectives of this study and underscore barriers for older adults related to digital literacy, usability, and accessibility [18]. Addressing these challenges requires solutions that combine technical robustness with user-centered design principles tailored to ageing populations.

In response, this work presents Rehabi, a friendly mHealth telerehabilitation application that integrates a markerless motion-capture system with dedicated clinician and patient interfaces to support remote rehabilitation. The system combines offline-first functionality, personalized exercise programs, and markerless motion analysis to enable intuitive, bidirectional interaction while reducing digital-literacy barriers through accessible design and clear feedback. Pilot clinical testing explored the feasibility of objectively monitoring hip and knee range of motion by comparing markerless measurements against professional assessments using the Intraclass Correlation Coefficient (ICC). Pilot study data (*n* = 14) suggest moderate reliability for hip range of motion (ICC = 0.686) and good reliability for knee range of motion (ICC = 0.801). With an initial focus on postoperative care following total hip and knee arthroplasty, Rehabi couples practical usability with explicit biomechanical angle estimation and resilient synchronization for low-connectivity settings, highlighting the potential of markerless motion assessment as a feasible tool for routine clinical follow-up.

## 2. Materials and Methods

The development of Rehabi followed a user-centered design methodology, ensuring that both patient and clinician perspectives guided the system architecture and implementation. The methodological process was structured into four sequential phases: (i) requirements elicitation, (ii) system design, (iii) implementation, and (iv) clinical testing of the video-based motion analysis module.

### 2.1. Functional and Non-Functional Requirements Elicitation

To define the functional requirements, understood as the specific actions and behaviors a software system must perform to meet user needs, the study employed a structured questionnaire designed in accordance with established practices in requirements engineering [30]. All items in the questionnaire were formulated as open-ended questions and organized into four thematic sections: (i) patient usability and accessibility, (ii) clinician requirements for monitoring and prescription, (iii) technical feasibility in terms of device availability and connectivity, and (iv) interface preferences, including layout and color schemes. Because the questionnaire was conceived as an exploratory tool for requirements elicitation rather than as a standardized measurement scale, a full psychometric validation was not undertaken. Instead, its adequacy was supported by operational validation processes inherent to software development, including iterative prototyping.

The questions grouped in each section, together with their respective purposes, are presented in Table 1. The questionnaire was distributed among patients undergoing musculoskeletal rehabilitation and medical professionals supervising therapy. No personal identification data was collected, and all participants provided consent to a privacy statement before participation. Responses were further complemented by semi-structured interviews, which provided qualitative insights into barriers, expectations, and desired functionalities.

Because all the questionnaire items were open-ended, interpreting responses was challenging. To address this, natural language processing (NLP) techniques were applied to support the analysis of textual data. Data analysis was conducted in three stages using Python libraries (Pandas 2.2.2, NumPy 1.26.4, Matplotlib 3.8.4, scikit-learn 1.5.1, and the Natural Language Toolkit 3.9.1), as well as NLP methods. Stage 1 focused on data cleaning, including removing incomplete, duplicate, or inconsistent responses and converting categorical variables. Stage 2 involved descriptive statistical analysis, primarily applied to sociodemographic variables and closed-ended questions. Stage 3 implemented NLP-based thematic modeling to identify recurring themes, clusters of needs, and user expectations derived from the open-ended responses.

For stage 3, all textual responses were processed in Spanish using spaCy’s (version 3.8.0) “es_core_news_sm model” to support thematic analysis. The preprocessing pipeline included tokenization and lemmatization; removal of stopwords and non-alphabetic tokens; and exclusion of tokens whose POS tags belonged to the set [‘CONJ’, ‘ADP’, ‘DET’]. The remaining tokens were converted to lowercase lemmas and concatenated at the document level. A CountVectorizer(scikit-learn version 1.8.0) was then applied to the lemmatized corpus to construct a document–term matrix and a lemma-based vocabulary. Term frequencies derived from this vocabulary were used to generate a top-term bar chart and a word cloud, ensuring that the thematic analysis relied on normalized lexical forms rather than surface-level inflections.

### 2.2. System Design

The application was designed using a layered architecture that adheres to the principles of Clean Architecture. Layered architectures are considered a best practice in software engineering because they enforce modularity, scalability, and maintainability [31,32].

Two types of layered architecture are commonly described in the literature: rigid architectures, which enforce strict top-down communication, and flexible architectures, which allow lower layers to communicate with upper layers when necessary. For this project, a flexible layered architecture was selected to handle multimedia components, including video and real-time image processing [33].

**Figure 1:**
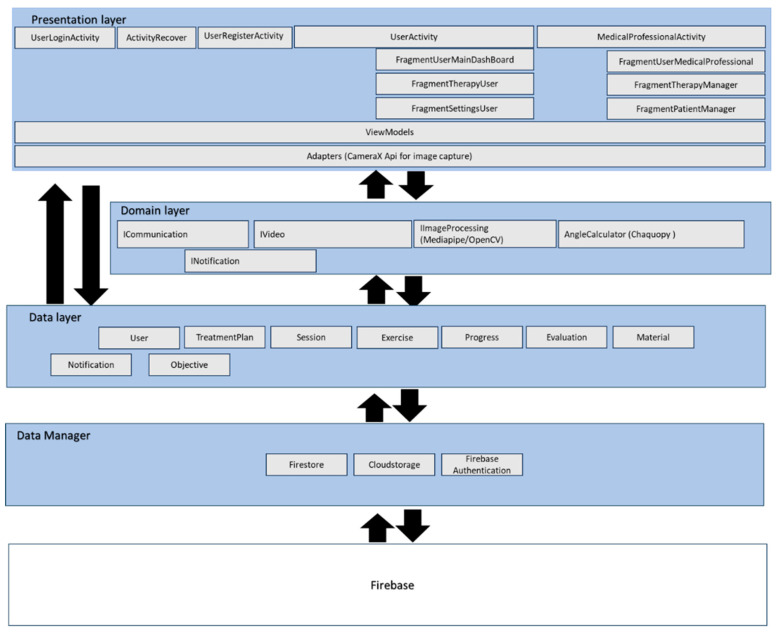
Layered architecture of the telerehabilitation application showing Presentation, Domain, and Data layers with their respective components and data flow.

The architecture was divided into three layers. The Presentation Layer, which is responsible for the user interface, is implemented in Kotlin and XML using Android Studio (Ladybug Feature Drop 2024.2.2). It contains Activities, Fragments, ViewModels, and adapters that manage UI logic and user interaction. The Domain Layer contains the business logic and use cases, designed to be independent of Android and external frameworks. This layer defines a set of interfaces, such as ICommunication, INotification, IVideo, and IImageProcessing, that serve as abstraction points between the core logic and external dependencies. These interfaces enable loose coupling, making the system more flexible, testable, and maintainable by allowing the implementation details to be swapped or extended without modifying the core logic. The application’s layered design architecture is shown in Figure 1.

Crucially, external libraries used for image processing and pose detection, MediaPipe (com.google.mediapipe:tasks-vision:0.10.21) [34], OpenCV (org.opencv:opencv:4.9.0) [35], and Chaquopy (15.0.1) [36], were accessed through these interfaces. This design ensures that the Domain Layer does not depend on these libraries directly; instead, it interacts with them through well-defined contracts. For example, pose estimation functions from MediaPipe and OpenCV were encapsulated behind the interface, allowing the business logic to request movement analysis without being tied to a specific implementation. For example, within the Presentation Layer, camera frames are acquired using Android’s CameraX API and converted into bitmaps. These frames are passed through an implemented IImageProcessing interface, which abstracts away the details of MediaPipe/OpenCV, so that the Domain Layer does not depend directly on external libraries.

The Domain Layer then applies the biomechanical logic encapsulated in the AngleCalculator class. This component constructs a simplified skeletal model of the lower limb using hip, knee, ankle, and toe landmarks from MediaPipe, which already provide normalized coordinates. In addition, missing landmarks were handled by using linear interpolation, in which the last valid coordinate was connected to the next available coordinate by a straight line. Intermediate frames were filled by proportionally distributing values along this line, ensuring smooth, continuous motion without abrupt jumps. On top of this skeletal representation, the AngleCalculator implements reusable mathematical routines for joint angle estimation, defined entirely in terms of vectors and dot products (see Equation (1)).

The joint angles are derived from landmark vectors using the dot-product formula:

(1) $$\theta_{\deg}=\arccos\left(\frac{\vec{u}\cdot \vec{v}}{\left||\vec{u}|||\;|\vec{v}|\right|}\right)\cdot \frac{180}{\pi }$$

where

$\vec{u}\cdot \vec{v}$ is the dot product of the two vectors. It measures how much the vectors point in the same direction and $\left||\vec{u}|||\;|\vec{v}|\right|$ are the magnitudes (lengths) of the vectors. Based on this formulation:

- Hip angle: computed between the thigh vector (Hip → Knee) and the vertical axis (0, −1). This quantifies the extent to which the thigh deviates from upright posture.
- Knee angle: computed between the thigh vector (Hip → Knee) and the shank vector (Knee → Ankle). This yields the flexion/extension of the knee joint.
- Ankle angle: computed between the shank vector (Knee → Ankle) and the foot vector (Ankle → Toe). This represents dorsiflexion/plantarflexion at the ankle.

**Figure 2:**
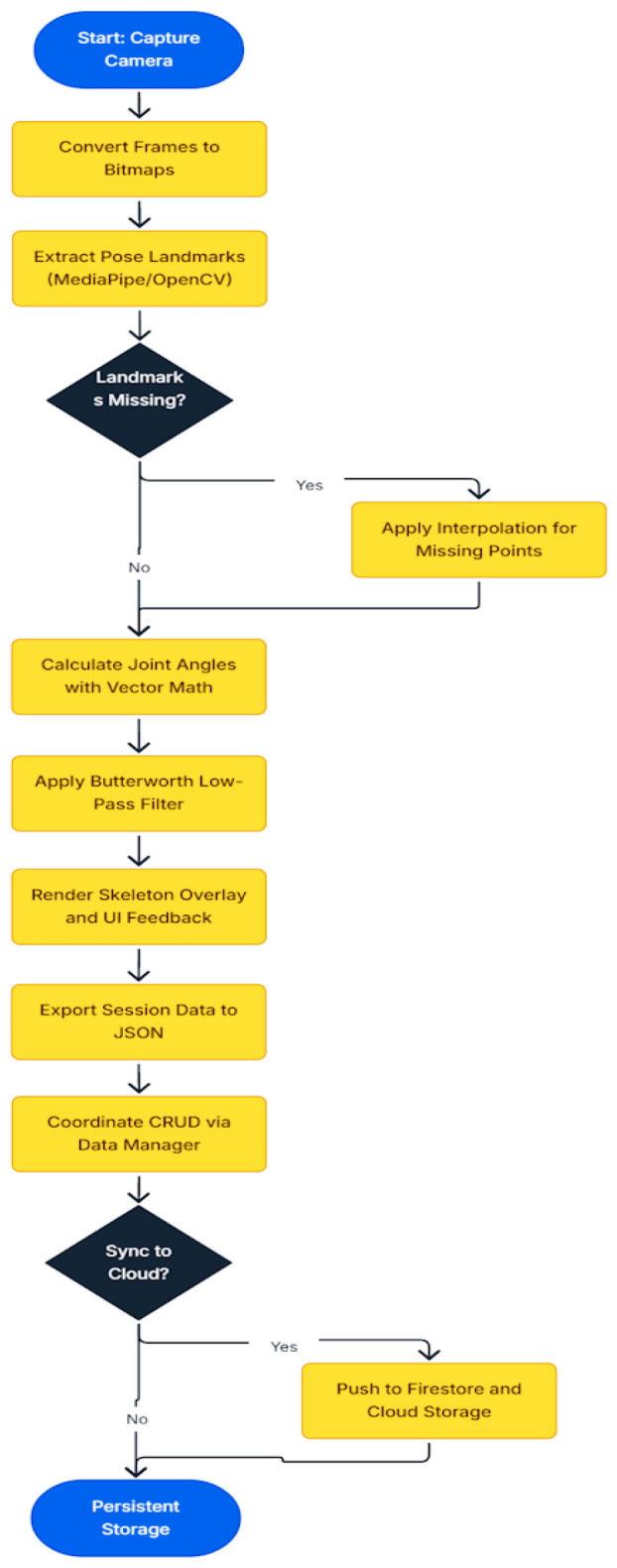
A flowchart that summarizes how data flows for the markerless motion analysis algorithm.

Each angle is calculated per frame, converted to degrees, and logged with a timestamp. To improve robustness, the vectors are normalized, the dot product is clamped to [−1, 1] to avoid numerical errors, and a 4th-order Butterworth low-pass filter with a 6 Hz cutoff frequency is applied to the calculated joint angles, reducing frame-to-frame jitter and attenuating high-frequency noise while preserving physiologically meaningful motion [37]. The resulting angles are then drawn directly on the skeleton overlay in the Presentation Layer, providing immediate visual feedback. They are exported as JSON for downstream analysis (see Figure 2 for the markerless motion flowchart algorithm).

The Data Layer manages data access and persistence. To ensure modularity and adaptability, this layer was implemented by creating a dedicated model for each database table, representing entities such as patients, medical professionals, treatment plans, sessions, exercises, progress records, evaluations, and notifications. Each model encapsulates the structure and attributes of its corresponding table, providing a clear mapping between the application logic and the underlying database schema.

In addition to the models, a Data Manager component was designed to integrate and intercept operations across repositories. The manager acts as a centralized controller, coordinating data flow between the application and Firebase services. It ensures that CRUD (Create, Read, Update, Delete) operations are consistently applied and handles synchronization between local storage and remote sources. This design allows the system to enforce validation rules, manage transactions, and apply business constraints before data is persisted.

The Data Manager interacts with Firebase components in a modular way:

• Firebase Firestore [38] is used for structured database management and offline-first synchronization.
• Firebase Cloudstorage [39] handles secure multimedia storage, such as exercise videos or patient images.
• Firebase Authentication [40] manages authentication and user security.

By combining table-specific models with a centralized manager, the Data Layer achieves a balance between flexibility and control.

### 2.3. Interface Design

Because the application required a dual-user model, the system was designed with two distinct interfaces tailored to the needs of medical professionals and patients. The medical professional interface enables uploading instructional videos, prescribing individualized exercise sets and repetitions, and monitoring patient progress through dashboards and evaluation tools. The patient interface allows users to access prescribed exercises, record their performance using the device’s camera, and receive real-time feedback based on pose detection and biomechanical analysis powered by MediaPipe and OpenCV.

The interface design emphasizes clarity and accessibility, particularly for older adults recovering from hip arthroplasty. Visual specifications were carefully selected to reduce cognitive and visual load: a green-based color palette was adopted for its calming and therapeutic qualities, consistent with psychological literature highlighting green tones as relaxing [41]. Contrast-enhanced combinations (dark green with white text, black on light pastel backgrounds) ensure legibility for users with age-related visual impairments.

Typography is a critical factor in usability, particularly for older adults. The application employs Roboto and Open Sans, two sans-serif typefaces widely recognized for their clarity and optimization in mobile environments. To enhance legibility, font sizes were deliberately increased beyond standard Android defaults: a minimum of 18 sp for body text (≈13.5 pt), 20 sp for subtitles (≈15 pt), and 24 sp for headers (≈18 pt). These values are significantly larger than the conventional 12-point print size, aligning with recommendations in design literature for older adults [42], which emphasizes the importance of larger, high-contrast fonts to reduce visual strain and improve readability.

Interaction mechanics were inspired by familiar platforms such as Netflix (for intuitive content delivery) and WhatsApp (for simplified communication), ensuring that users engage with the system in a natural, stress-free manner. This dual-interface strategy, combined with modular architecture and real-time feedback mechanisms, reinforces the system’s clinical relevance, usability, and scalability for telerehabilitation scenarios.

### 2.4. Mobile App Video-Based Motion Analysis Testing

To validate the proposed video-based motion analysis system in a clinical context, a pilot study was conducted at the National Institute of Rehabilitation Luis Guillermo Ibarra Ibarra (INRLGII) to determine whether markerless motion recordings could reliably capture clinically relevant hip and knee flexion in post-arthroplasty patients. Patients were invited to participate immediately after their clinical consultation, which limited recruitment because relatively few individuals met the inclusion criteria and agreed to enroll; consequently, the initial cohort comprised 14 participants (9 THA, 5 TKA). Eligible participants were aged ≥18 years, at least six weeks post-surgery, and able to complete standardized functional tests; individuals with severe comorbidities, cognitive impairment, or physical limitations that could compromise safe participation were excluded. Comparative assessments were performed by rehabilitation specialists trained in goniometry and motion analysis; to reduce within-subject variability, each exercise was repeated three times, and the mean value was used for analysis.

Functional recovery was evaluated by measuring hip and knee flexion: hip flexion was assessed in the supine position with the operated limb raised toward the chest, and knee flexion was measured in both seated and supine positions to capture the maximal range of motion. Video recordings were acquired using a Lenovo Tab M11 tablet (MediaTek Helio G88, 8 GB RAM, 128 GB storage) with a 13-megapixel rear camera; an operator hand-held the device at an approximate height of 1.50 m and a distance of 2.5 m from the participant to ensure full-body visibility. Recordings were captured at 1080 p resolution and 15–20 Hz, under diffuse, even lighting and a neutral background, to minimize landmark-detection errors. Pose estimation was implemented using the MediaPipe Pose Landmarker full model. This configuration enabled robust anatomical landmark detection and reliable estimation of hip, knee, and ankle joint angles during the rehabilitation exercises.

Clinical goniometric measurements were performed by two board-certified physicians specializing in Physical and Rehabilitation Medicine, each with advanced subspecialty training in Orthopedic Rehabilitation and more than five years of experience managing arthroplasty patients. Patients with total hip arthroplasty were evaluated by one specialist with extensive experience in hip arthroplasty rehabilitation. In contrast, patients with total knee arthroplasty were evaluated by a different specialist with equivalent expertise in knee arthroplasty rehabilitation. Both evaluators were blinded to the markerless motion analysis system results, and clinical measurements were performed before algorithm-based analysis to ensure independence of the reference standard and minimize potential measurement bias. Quantitative measures derived from the markerless algorithm were therefore compared with this gold-standard clinical goniometry performed by trained clinicians within a standardized, reproducible pipeline that minimized observer bias through experienced raters and consistent procedures.

Agreement and reliability between clinical goniometry and markerless motion analysis were assessed using intraclass correlation coefficients (ICCs) with 95% confidence intervals and Bland–Altman plots to evaluate systematic bias and the limits of agreement. ICCs were calculated using a two-way mixed-effects model with absolute agreement (ICC(A,1)) and 95% confidence intervals to quantify the precision of the estimates. All ICC calculations were performed in R (R Foundation for Statistical Computing, Vienna, Austria, version 4.5.2).

## 3. Results

### 3.1. Requirements Determination

A total of 34 respondents participated in the requirements-elicitation phase: 20 medical professionals (physicians, biomedical engineers, nurses, and therapists) and 14 patients undergoing musculoskeletal rehabilitation. Most respondents were medical professionals, predominantly women aged 31–40 years, and their feedback revealed several recurring themes that shaped the application’s requirements.

In terms of needs and expectations, participants emphasized the importance of video consultations, flexible scheduling, patient monitoring, and interactive tutorials. At the same time, they identified significant barriers, particularly the limited digital literacy among older adults and the reliance on continuous internet connectivity, which remains a challenge in many contexts. When asked about preferred functionalities, respondents highlighted video-based therapy, reminders and notifications, progress tracking, and direct communication with clinicians as essential features. Their evaluation criteria focused on ease of use, functionality, speed, and low dependence on internet connectivity. To support onboarding and troubleshooting, tutorials and video guides were the most requested mechanisms.

These findings informed the definition of the application’s functional requirements (e.g., registration, video communication, therapy assignment, progress tracking, notifications) and non-functional requirements (e.g., usability, accessibility, offline capabilities).

As mentioned in Section 2.1 (Functional and non-functional requirements elicitation), the non-functional requirements of the proposed application were defined through a review of recent systematic and comparative studies on telemedicine and telerehabilitation, which emphasize the importance of ethical safeguards, usability in low-resource contexts, and technological reliability. From this analysis, three critical priorities were highlighted: (i) information security, ensuring confidentiality and compliance with ethical standards, as underscored in reviews addressing governance, legal frameworks, and data privacy in telehealth [1,2] (ii) offline processing and storage, guaranteeing functionality in rural or low-connectivity environments, a need repeatedly mentioned in studies of telerehabilitation in developing countries and in pandemic contexts [2,43], and (iii) synchronization mechanisms, enabling secure data transfer once connectivity is restored, reflecting recommendations in telemedicine applications for hemophilia and stroke monitoring, where intermittent reporting and delayed uploads are common [8]. These requirements were essential to align the application with the realities of healthcare delivery in Mexico and other low and middle-income countries, where internet access remains uneven.

### 3.2. Proposed Developed Application

**Figure 3:**
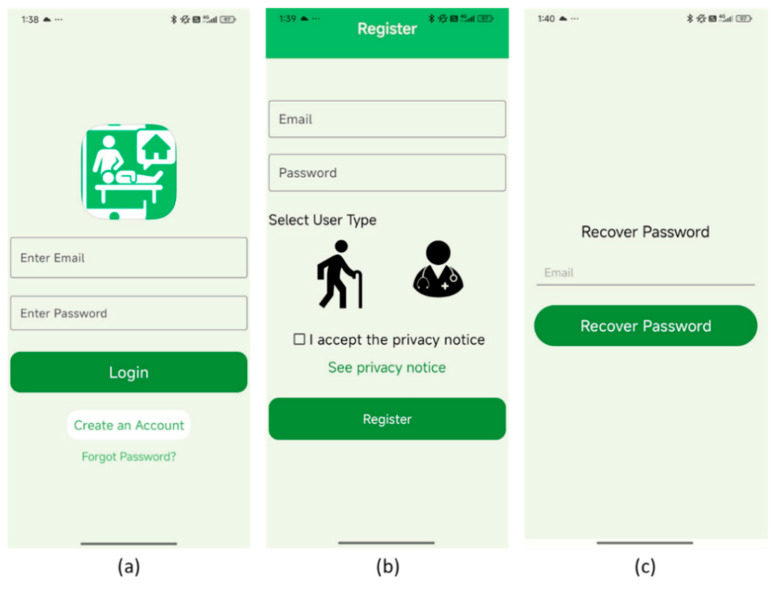
Authentication interfaces of the mobile application, including login, registration, and password recovery screens. (**a**) Login screen, (**b**) Registration screen, (**c**) recovery password screen.

Figure 3 shows the authentication interface of the mobile application, comprising three core screens: login (Figure 3a), registration (Figure 3b), and password recovery (Figure 3c). The login screen (Figure 3a) includes input fields for email and password, a green “Login” button, and navigation options for account creation and password reset, all framed by a healthcare-themed icon that reinforces the app’s clinical context. The registration screen (Figure 3b) allows users to enter credentials, select their role (patient or healthcare professional) via intuitive icons, and accept the privacy notice before submitting. The password recovery screen (Figure 3c) is minimal, prompting users to enter their email address to initiate a reset. Together, these interfaces demonstrate a user-centered design that supports secure access, role differentiation, and usability across varying levels of digital literacy.

**Figure 4:**
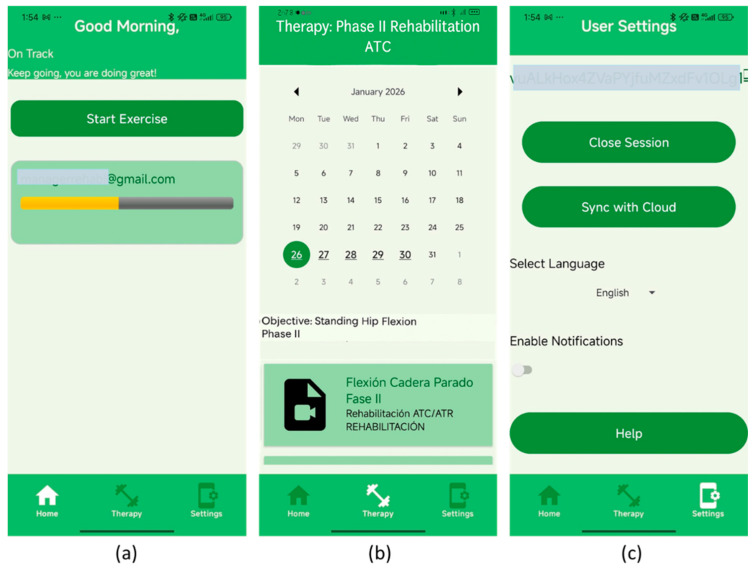
Patient interface of the rehabilitation application, showing dashboard (**a**), therapy calendar (**b**), and settings panel (**c**). These screens illustrate a user-centered design that supports accessibility, secure access, and personalized rehabilitation tracking.

Figure 4 illustrates the patient-facing interface of the rehabilitation application, structured around three core screens: the main dashboard (Figure 4a), the therapy calendar (Figure 4b), and the settings panel (Figure 4c). Each module is designed to enhance rehabilitation tracking, accessibility, and patient engagement.

The main dashboard (Figure 4a) welcomes the user with motivational prompts (e.g., “Good Morning” and “Keep going, you are doing great!”), reinforcing adherence and progress. A green Start Exercise button provides direct access to the day’s assigned routine, while a progress bar beneath the user’s email tracks completion status. A bottom navigation menu (Home, Therapy, Settings) ensures quick access to core modules. The therapy calendar (Figure 4b) presents a monthly view with highlighted dates for scheduled sessions. Selecting a date clarifies the rehabilitation objective and the specific exercises, such as “Standing Hip Flexion—Phase II,” within the rehabilitation context. This design supports self-monitoring and continuity across therapy phases. The settings panel (Figure 4c), accessible to both patient types and medical professionals, provides session management and personalization tools. Options include Close Session, Sync with Cloud, language preferences, and notification settings. A Help button provides access to support resources, ensuring usability across varying levels of digital literacy.

**Figure 5:**
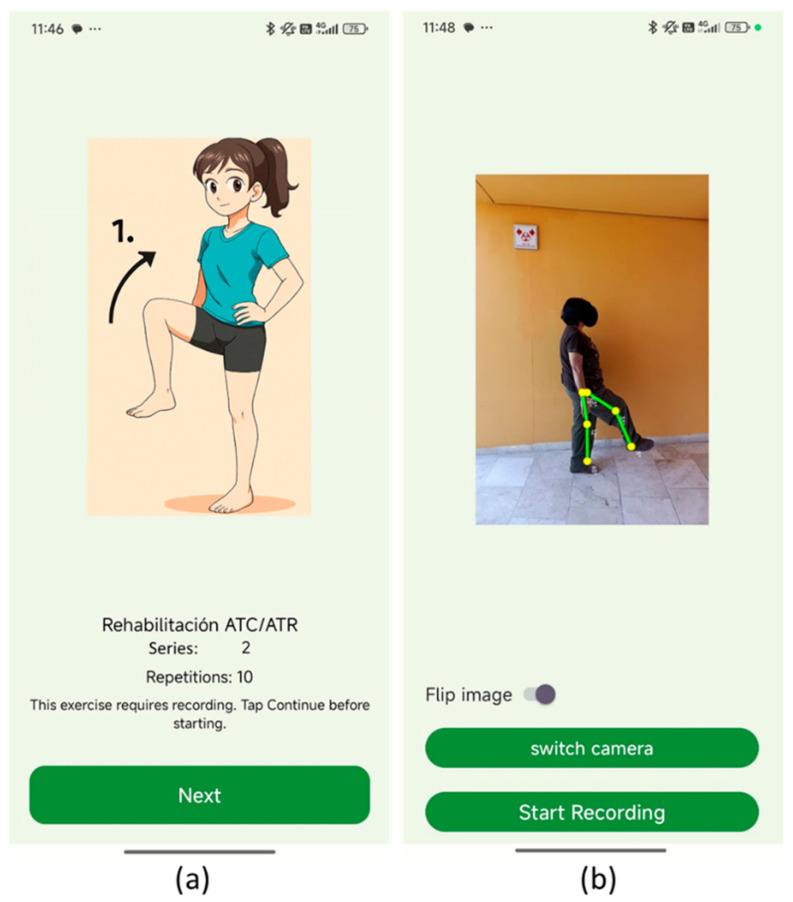
Application screens for rehabilitation session initialization. (**a**) Exercise demonstration screen. (**b**) Active performance and recording screen.

At the beginning of each rehabilitation session, the proposed application presents two sequential interfaces that integrate instructional guidance with real-time monitoring, as shown in Figure 5. The first screen, Figure 5a, provides a continuous demonstration of the assigned exercise, such as a knee lift, via a looping video. This ensures that the patient clearly understands the movement pattern before performing the assigned exercise. The interface also specifies the number of sets and repetitions prescribed for the exercise, reinforcing adherence to the rehabilitation protocol. The demonstration remains active until the patient selects the Next button, allowing them to proceed at their own pace. The second screen (Figure 5b) transitions to active performance and recording. The patient is captured on the device’s camera while executing the exercise. A biomechanical overlay is generated in real time, marking the hip, knee, and ankle joints and displaying the corresponding anatomical angles. This visualization provides immediate feedback to the patient and enables clinicians to later evaluate the quality of movement. Patients may record independently using a tripod or request assistance from a caregiver. Additional interface elements include a toggle to flip the camera view and buttons to switch between front and rear cameras or to initiate recording. Once activated, the system collects exercise data, automatically stores goniometric measurements in a structured JSON format, and uploads them to the cloud.

**Figure 6:**
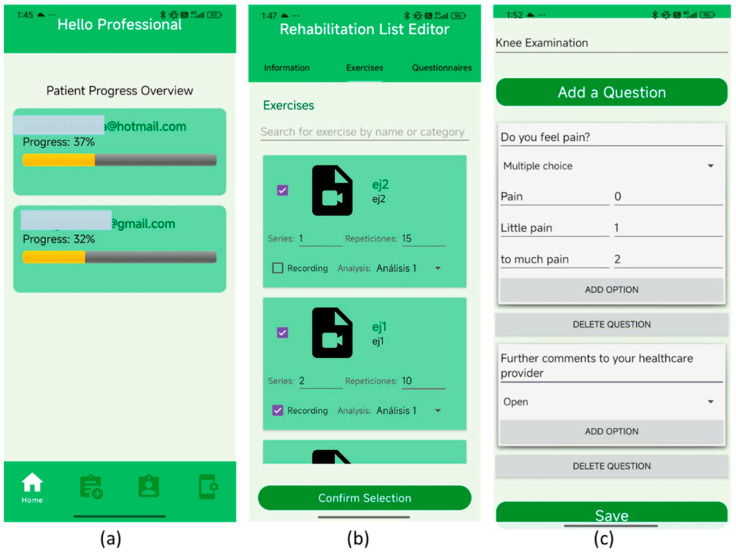
Professional user interface showing (**a**) main dashboard for monitoring patient progress, (**b**) personalized therapy editor with exercise assignment, repetitions, and optional motion-tracking recording, and (**c**) custom questionnaire creation system for individualized therapy evaluation.

Figure 6 presents the medical professional user interface of the rehabilitation application, composed of three functional screens tailored for clinical monitoring and customization. Figure 6a shows the main dashboard, which provides an overview of patient progress, displaying individual completion percentages through color-coded progress bars, allowing clinicians to track adherence and recovery trends at a glance. Figure 6b shows the personalized therapy editor screen, which enables healthcare professionals to assign specific exercises to each patient, define the number of sets and repetitions, and, optionally, activate motion tracking via the “Recording” checkbox for video-based analysis. Figure 6c shows that the custom questionnaire module supports individualized therapy evaluation by allowing the creation of open-ended and multiple-choice questions, assigning scores to each response, and enabling automated assessment during patient sessions. Together, these components facilitate precise therapy planning, real-time monitoring, and adaptive evaluation within a unified clinical workflow.

### 3.3. Mobile App Video-Based Motion Analysis Results

A total of 14 patients participated in the clinical validation phase. The cohort included individuals with either THA or TKA. The average time since surgery was 7.14 months (range, 1–60 months). Surgical distribution comprised nine patients with hip arthroplasty (including bilateral cases) and five with knee arthroplasty. Table 2 summarizes the demographic and clinical characteristics of the cohort, including age, body metrics, surgical site, and time since surgery.

**Figure 7:**
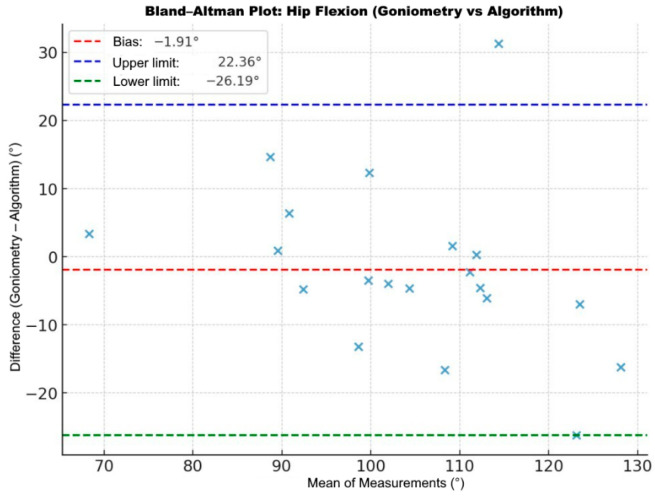
Bland–Altman plot comparing hip flexion measurements between clinical goniometry and the app algorithm. The ‘×’ markers represent individual paired data points, with the mean bias and limits of agreement shown as dashed lines.

Intraclass correlation analysis demonstrated moderate-to-good agreement between clinical goniometry and the markerless motion analysis system. For hip flexion, the ICC was 0.686 (95% CI: 0.362–0.863, *p* = 0.00029). Bland–Altman analysis revealed a mean bias of −1.91°, with limits of agreement of ±22.36° and ±26.19° (Figure 7).

**Figure 8:**
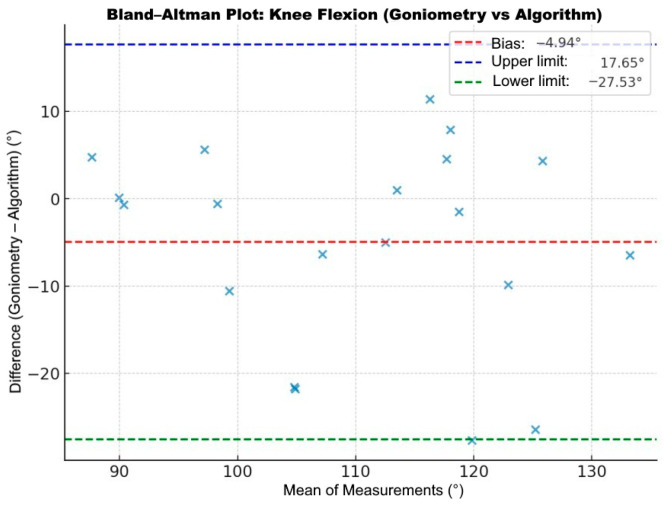
Bland–Altman plot comparing knee flexion measurements between clinical goniometry and the app algorithm. The ‘×’ markers represent individual paired data points, with the mean bias and limits of agreement shown as dashed lines.

For knee flexion, the ICC was 0.801 (95% CI: 0.571–0.916, *p* < 0.001). Bland–Altman analysis indicated a mean bias of −4.94°, with limits of agreement of ±17.65° to ±27.53° (Figure 8).

**Figure 9:**
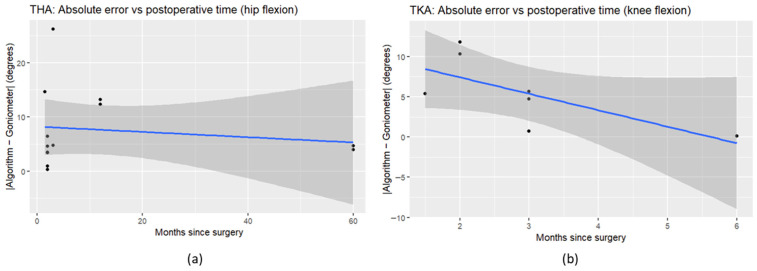
Postoperative time analysis in THA (**a**) and TKA (**b**) patients, ilNLP-based requirements.

In the THA subgroup, postoperative time was not associated with absolute measurement error for hip flexion (β = −0.049° per month, *p* = 0.637; R^2^ = 0.021); similarly, postoperative time was not associated with signed differences (β = 0.055° per month, *p* = 0.715). Spearman’s correlation indicated no significant association between postoperative time and absolute error (ρ = 0.212, *p* = 0.487), while a non-significant trend was observed for signed differences (ρ = 0.540, *p* = 0.056). In the TKA subgroup, postoperative time showed a non-significant trend toward reduced absolute measurement error for knee flexion (β = −2.047° per month, *p* = 0.0837; R^2^ = 0.482); no association was observed for signed differences (β = −1.049° per month, *p* = 0.648). Spearman correlation similarly suggested a non-significant negative association between postoperative time and absolute error (ρ = −0.692, *p* = 0.0847). Given the limited sample size in the TKA subgroup (*n* = 7), these findings are exploratory and should be interpreted with caution; confirmation in larger, adequately powered cohorts is warranted (see Figure 9 for operative time results plots).

Overall, the algorithm proved valid for knee flexion in both hip and knee arthroplasty patients, while hip flexion results showed moderate validity. These findings highlight the feasibility of integrating markerless motion analysis into telerehabilitation platforms and identify areas for refinement, particularly in contracture assessment. The results emphasize the importance of protocol optimization to ensure clinical reliability in diverse rehabilitation contexts.

## 4. Discussion

The NLP-based requirements-elicitation process presents several limitations that must be considered when interpreting the findings. Recruitment on demand introduced potential selection bias, and the modest, uneven sample, combined with open-ended items, means that thematic frequenis should be viewed as exploratory rather than definitive. The qualitative depth of the interviews partially offset these constraints, as did the direct involvement of medical professionals and continuous collaboration with the software development team, which enriched interpretation beyond the NLP-derived themes. Although the questionnaire was intentionally designed as an exploratory tool, future work could incorporate psychometric validation to strengthen reliability. Nonetheless, in software engineering practice, early requirement gathering commonly relies on stakeholder experience, iterative feedback, and developer expertise, making this approach appropriate for an initial design cycle. The integration of clinical insight with iterative development aligns naturally with agile methodologies, supporting continuous refinement and responsiveness to evolving user needs.

Rehabi’s architecture follows Clean Architecture principles, and its modular layer separability provides a reusable blueprint for other mHealth applications by enabling sensor-agnostic processing, offline-first synchronization, and a clear separation between domain logic and device-specific implementations. By abstracting image-processing components, storing calibration metadata, and exporting reproducible preprocessing parameters, the platform strengthens reproducibility and testability.

The pilot clinical validation of the markerless motion algorithm demonstrated preliminary feasibility for mobile markerless motion analysis in post-arthroplasty patients. In a cohort of 14 participants, hip flexion showed moderate agreement (ICC = 0.687), while knee flexion demonstrated good agreement (ICC = 0.803). These results support the feasibility of mobile markerless assessment, yet they should be interpreted as early-stage findings requiring confirmation in larger, controlled studies. Differences from the higher ICCs reported in the literature (0.81–0.98 for spatiotemporal and goniometric metrics [22,23,24]) can be attributed to methodological heterogeneity, including variations in capture hardware, sampling frequency, camera geometry, operator technique, pose-model conventions, signal-processing pipelines, and the clinical reference standard.

Importantly, these results align with trends highlighted in the Introduction: the shift toward mHealth delivery, the growing use of AI-driven motion-tracking tools, and the emergence of markerless systems as practical, low-cost, and easy-to-use alternatives to traditional motion-capture technologies [16,17,21].

Complementary Bland–Altman analyses provided further insights into measurement error. Across the overall sample, hip flexion showed a mean bias of 1.91°, while knee flexion demonstrated a mean bias of 2.22°, indicating minimal systematic bias. However, the limits of agreement were relatively wide, particularly for hip flexion (−22.36° to 26.19°), suggesting moderate variability at the individual measurement level. Subgroup analyses revealed that THA patients exhibited minimal bias (1.77°) but wide limits of agreement and statistically significant proportional bias (*p* = 0.035), indicating reduced accuracy at higher degrees of flexion. In contrast, knee flexion in TKA patients demonstrated minimal bias (0.98°), narrower limits of agreement (−13.38° to 15.35°), and no proportional error (*p* = 0.136), supporting greater reliability. These findings suggest that markerless motion analysis could be clinically suitable for monitoring functional progression.

Rehabi’s findings, therefore, reinforce the broader evidence that mobile markerless motion analysis is promising but technically demanding. By implementing standardized capture protocols, per-session calibration, device and operator testing, and a powered multicenter validation with predefined acceptability thresholds, the platform can progress from a feasible prototype to a validated clinical tool. The architectural and methodological lessons reported here are broadly applicable and can inform the development and validation of other mHealth applications that rely on markerless motion analysis or similar sensing pipelines.

## 5. Conclusions

This work introduces Rehabi, a user-friendly mHealth telerehabilitation platform that combines accessible clinician and patient interfaces with integrated markerless motion analysis. In a pilot study of 14 post-arthroplasty patients (9 THA, 5 TKA), recordings produced good agreement for knee flexion (ICC = 0.801) and moderate agreement for hip flexion (ICC = 0.686). The platform’s principal contribution is the marriage of an accessible, clinician-driven workflow with real-time pose feedback (visual overlay and JSON export), which lowers barriers to remote assessment and supports patient engagement. These results are promising but preliminary: individual measurement variability, sensitivity to capture conditions, and the small sample size limit immediate clinical generalization. To advance Rehabi toward routine use, we recommend larger multicenter validation (target *n* > 100), per-exercise calibration, and standardized capture protocols.

## Figures and Tables

**Table 1:** Structure of User Questionnaire for Requirements Elicitation in the proposed application development.

Section	Questions	Purpose of Data Collected
1. Sociodemographic Information	• Please select your age range. • Please select your gender. • What is your profession/occupation? • What type of user would you be for the application? (e.g., patient, doctor, therapist, etc.)	To characterize the respondent population and classify user roles. This ensures that requirements reflect the diverse demographics and perspectives of both patients and clinicians.
2. Needs and Problems	• What problems do you expect this type of application to solve? • How do you think each of these problems should be solved in detail? • At present, do you know of any application, system, technology, or digital tool that solves the problems you mentioned above? • What functions do you expect to find in teleconsultation and telerehabilitation applications, and why/for what purpose?	To capture user pain points, expectations, and benchmarks against existing solutions. This informs the definition of functional requirements and highlights unmet needs.
3. User Environment and Technology Use	• Who do you think will be the main users of the system? • How well do you think you can use a computer or mobile device (smartphone, tablet, etc.)? • How well do the people around you know how to use a computer or mobile device (smartphone, tablet, etc.)? • Do you know of any system or mobile application that performs a function you like, and that you would like to see in a teleconsultation or telerehabilitation system? • Which mobile applications are easiest for you to use (any type, e.g., WhatsApp), and why? • When you use a mobile application for the first time, what do you think is the best way to learn how to use it? • When you use a mobile application, what do you think is the best way to receive help for its use?	To assess digital literacy, usability preferences, and support needs. This section outlines non-functional requirements, including accessibility, onboarding strategies, and help mechanisms, to ensure inclusivity and ease of adoption.
4. Evaluation of Opportunities	• What parameters or elements do you evaluate to determine whether a mobile application meets your needs? • Is there any feature you would like this type of application to include?	To understand user acceptance criteria and identify opportunities for innovation. This ensures the application aligns with user expectations and incorporates desirable features.

**Table 2:** Demographic and clinical characteristics of 14 post-Total Hip Arthroplasty (THA) or Total Knee Arthroplasty (TKA) patients, including age (years), weight (kilograms kg), height (meters m), surgery type, and time since surgery (months).

Age (Years)	Weight (kg)	Height (m)	Surgery Type	Months Since Surgery (Months)
61	78	1.63	Left THA	60
50	94	1.72	Left THA	2
64	61.5	1.48	Right TKA	2
73	69.8	1.56	Left TKA	1
48	67	1.54	Left and Right THA	2
72	72	1.89	Right TKA	2
66	58	1.57	Left TKA	3
68	68	1.63	Left THA	1.5
77	68.3	1.37	Right TKA	3
50	74	1.58	Left and Right THA	12
63	47.3	1.42	Left THA	3
55	78.5	1.5	Left THA	6
46	90	1.72	Left and Right THA	1
53	55	1.57	Left THA	1.5

## Data Availability

The original data presented in the study are openly available in RehabiData at https://github.com/canibulin/RehabiData (accessed on 3 March 2026).

## Abbreviations

The following abbreviations are used in this manuscript:

THA	Total Hip Arthroplasty
TKA	Total Knee Arthroplasty
ICC	Intraclass Correlation Coefficient
NLP	Natural Language Processing
MVIs	Medical Virtual Instruments
IoT	Internet of Things
IPAQ	International Physical Activity Questionnaire
GNSS	Global Navigation Satellite System
mHealth	Mobile Health
UI/UX	User Interface/User Experience
CRUD	Create, Read, Update, Delete
API	Application Programming Interface
AI	Artificial Intelligence
ICTs	Information Communication Technologies
